# 1-Ethyl-1-methyl-3-(2-nitro­benzo­yl)thio­urea

**DOI:** 10.1107/S1600536811024652

**Published:** 2011-06-30

**Authors:** Aisha A. Al-abbasi, Mohammad B. Kassim

**Affiliations:** a School of Chemical Sciences & Food Technology, Faculty of Science & Technology, Universiti Kebangsaan Malaysia, 43600 Selangor, Malaysia; b Fuel Cell Institute, Universiti Kebangsaan Malaysia, 43600 Selangor, Malaysia

## Abstract

In the title compound, C_11_H_13_N_3_O_3_S, the benzene ring is twisted relative to the amidic fragment, forming a dihedral angle of 27.26 (9)°. The thiono and carbonyl groups are *trans* with respect to the C—N bond. Inter­molecular N—H⋯S and C—H⋯O hydrogen bonds link the mol­ecules in the crystal structure.

## Related literature

For the synthesis, see: Al-abbasi *et al.* (2010 ▶). For related structures and background references, see: Shanmuga Sundara Raj *et al.* (1999 ▶); Arslan *et al.* (2003 ▶); Al-abbasi & Kassim (2011 ▶). For standard bond lengths, see: Allen *et al.* (1987 ▶) and for bond lengths in other substituted thio­ureas, see: Nasir *et al.* (2011 ▶); Pérez *et al.* (2011 ▶).
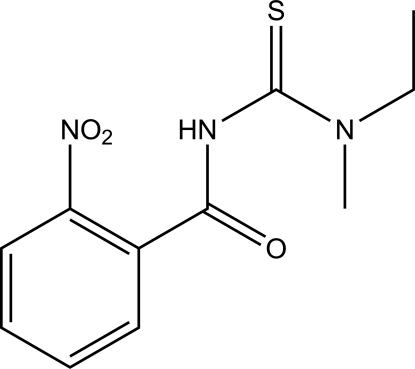


## Experimental

### 

#### Crystal data

C_11_H_13_N_3_O_3_S
*M* *_r_* = 267.30
Monoclinic, 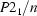

*a* = 11.447 (2) Å
*b* = 7.8664 (15) Å
*c* = 15.159 (3) Å
β = 107.128 (4)°
*V* = 1304.5 (4) Å^3^
*Z* = 4
Mo *K*α radiation
μ = 0.25 mm^−1^
*T* = 298 K
0.55 × 0.38 × 0.21 mm

#### Data collection

Bruker SMART APEX CCD area-detector diffractometer
Absorption correction: multi-scan (*SADABS*; Bruker, 2000 ▶) *T*_min_ = 0.874, *T*_max_ = 0.949
7105 measured reflections
2294 independent reflections
1971 reflections with *I* > 2σ(*I*)
*R*_int_ = 0.020

#### Refinement

*R*[*F* ^2^ > 2σ(*F* ^2^)] = 0.043
*wR*(*F* ^2^) = 0.124
*S* = 1.06
2294 reflections
169 parameters
1 restraint
H atoms treated by a mixture of independent and constrained refinement
Δρ_max_ = 0.33 e Å^−3^
Δρ_min_ = −0.19 e Å^−3^

### 

Data collection: *SMART* (Bruker, 2000 ▶); cell refinement: *SAINT* (Bruker, 2000 ▶); data reduction: *SAINT*; program(s) used to solve structure: *SHELXS97* (Sheldrick, 2008 ▶); program(s) used to refine structure: *SHELXL97* (Sheldrick, 2008 ▶); molecular graphics: *SHELXTL* (Sheldrick, 2008 ▶); software used to prepare material for publication: *SHELXTL*, *PARST* (Nardelli, 1995 ▶) and *PLATON* (Spek, 2009 ▶).

## Supplementary Material

Crystal structure: contains datablock(s) I, global. DOI: 10.1107/S1600536811024652/jh2299sup1.cif

Structure factors: contains datablock(s) I. DOI: 10.1107/S1600536811024652/jh2299Isup2.hkl

Supplementary material file. DOI: 10.1107/S1600536811024652/jh2299Isup3.cml

Additional supplementary materials:  crystallographic information; 3D view; checkCIF report

## Figures and Tables

**Table 1:** Hydrogen-bond geometry (Å, °)

*D*—H⋯*A*	*D*—H	H⋯*A*	*D*⋯*A*	*D*—H⋯*A*
N1—H1*A*⋯S1^i^	0.85 (2)	2.55 (2)	3.3828 (18)	167 (2)
C6—H6⋯O3^ii^	0.93	2.41	3.317 (3)	164

Symmetry codes: (i) 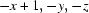; (ii) 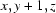.

**Fig. 1.**
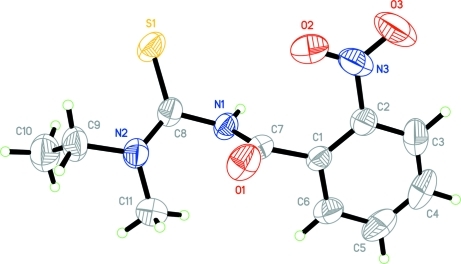
The molecular structure of the title compound with displacement ellipsoids drawn at the 50% probability level.

**Fig. 2.**
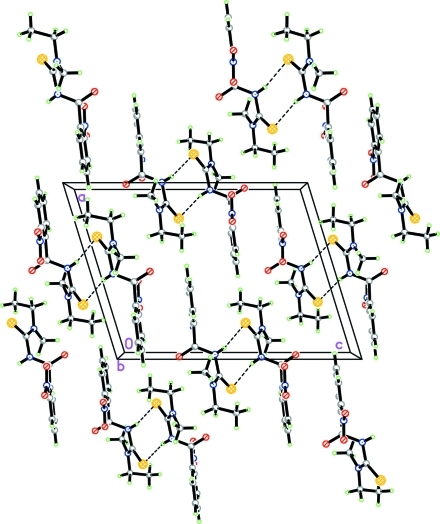
A packing diagram of the title compound viewed down the a-axis showing the intermolecular hydrogen bonds N1—H1A···S1 (-x + 1, -y, -z) and C6—H6···O3 (x, y + 1, z).

## supplementary crystallographic information

### Comment

The title compound, I, is a thiourea derivative analogous to our previously reported compounds (Al-abbasi & Kassim, 2011). Bond distances are similar to those usually found in other substituted thioureas [Nasir *et al.* (2011) & Pérez *et al.* (2011)]. The C–S and C–O exhibited the expected double-bond character. However, the C–N bond lengths are intermediate between a single and double, indicating a partial electron delocalization in the O1/C7/N1/C8/S1 fragment.

The phenyl ring is twisted due to the presence of the nitro group (O2O3N3) in *ortho* position. A rotation around C1—C7 bond makes the oxygen atom (O1) perpendicular to the phenyl ring mean planes and the torsion angles of C2C1C701 and C6C1C701 are -95.5 (2) and 86.5 (2)°, respectively. The dihedral angle between the mean planes of the thiourea (S1/N1/N2/C8/C9) and the phenyl ring (C1/C2/C3/C4/C5/C6/) plane is 27.56 (10)°. Other bond lengths and angles are in normal ranges (Allen *et al.* 1987).

The crystal structure is stabilized by the intermolecular N1—H1A···S1 and C5—H5A···O3 hydrogen bonds linking the molecules into a dimer resulting in a channel along [101] (Fig. 2).

### Experimental

The title compound was prepared according to a previously reported procedure (Al-abbasi *et al.*, 2010). A very pale browon colour crystal, suitable for X-ray crystallography, was obtained by a slow evaporation from ethanol solution at room temperature (yield 78%).

### Refinement

Hydrogen atom of the amide group was determined from the diffrence Fourrier map and N—H was initially fixed at 0.86(0.01) Å and allowed to be refined on the parent N atom with *U*_iso_(H) = 1.2*U*_eq_(N). All other H atoms were postioned geometrically with C—H bond lengths in the range 0.93 - 0.97 Å and refined in the riding model approximation with *U*_iso_(H)=1.2*U*_eq_(C,*N*), except for methyl group where *U*_iso_(H)= 1.5*U*_eq_(C).

### Crystal data

**Table:**

C_11_H_13_N_3_O_3_S	*F*(000) = 560
*M**_r_* = 267.30	*D*_x_ = 1.361 Mg m^−^^3^
Monoclinic, *P*2_1_/*n*	Mo *K*α radiation, λ = 0.71073 Å
Hall symbol: -P 2yn	Cell parameters from 4015 reflections
*a* = 11.447 (2) Å	θ = 2.0–25.0°
*b* = 7.8664 (15) Å	µ = 0.25 mm^−^^1^
*c* = 15.159 (3) Å	*T* = 298 K
β = 107.128 (4)°	Block, brown
*V* = 1304.5 (4) Å^3^	0.55 × 0.38 × 0.21 mm
*Z* = 4	

### Data collection

**Table:**

Bruker SMART APEX CCD area-detector diffractometer	2294 independent reflections
Radiation source: fine-focus sealed tube	1971 reflections with *I* > 2σ(*I*)
graphite	*R*_int_ = 0.020
ω scans	θ_max_ = 25.0°, θ_min_ = 2.0°
Absorption correction: multi-scan (*SADABS*; Bruker, 2000)	*h* = −13→13
*T*_min_ = 0.874, *T*_max_ = 0.949	*k* = −7→9
7105 measured reflections	*l* = −15→18

### Refinement

**Table:**

Refinement on *F*^2^	Primary atom site location: structure-invariant direct methods
Least-squares matrix: full	Secondary atom site location: difference Fourier map
*R*[*F*^2^ > 2σ(*F*^2^)] = 0.043	Hydrogen site location: inferred from neighbouring sites
*wR*(*F*^2^) = 0.124	H atoms treated by a mixture of independent and constrained refinement
*S* = 1.06	*w* = 1/[σ^2^(*F*_o_^2^) + (0.065*P*)^2^ + 0.531*P*] where *P* = (*F*_o_^2^ + 2*F*_c_^2^)/3
2294 reflections	(Δ/σ)_max_ < 0.001
169 parameters	Δρ_max_ = 0.33 e Å^−^^3^
1 restraint	Δρ_min_ = −0.19 e Å^−^^3^

### Special details

**Table:**

Geometry. All e.s.d.'s (except the e.s.d. in the dihedral angle between two l.s. planes) are estimated using the full covariance matrix. The cell e.s.d.'s are taken into account individually in the estimation of e.s.d.'s in distances, angles and torsion angles; correlations between e.s.d.'s in cell parameters are only used when they are defined by crystal symmetry. An approximate (isotropic) treatment of cell e.s.d.'s is used for estimating e.s.d.'s involving l.s. planes.
Refinement. Refinement of *F*^2^ against ALL reflections. The weighted *R*-factor *wR* and goodness of fit *S* are based on *F*^2^, conventional *R*-factors *R* are based on *F*, with *F* set to zero for negative *F*^2^. The threshold expression of *F*^2^ > σ(*F*^2^) is used only for calculating *R*-factors(gt) *etc*. and is not relevant to the choice of reflections for refinement. *R*-factors based on *F*^2^ are statistically about twice as large as those based on *F*, and *R*- factors based on ALL data will be even larger.

### Fractional atomic coordinates and isotropic or equivalent isotropic displacement parameters (Å^2^)

**Table:**

	*x*	*y*	*z*	*U*_iso_*/*U*_eq_	
S1	0.69163 (5)	0.06431 (8)	0.07382 (4)	0.0575 (2)	
O1	0.49702 (13)	0.2128 (2)	0.24893 (10)	0.0608 (4)	
O2	0.44396 (18)	−0.1541 (2)	0.17973 (16)	0.0811 (6)	
O3	0.2858 (2)	−0.3156 (2)	0.14610 (17)	0.0984 (7)	
N1	0.48480 (14)	0.1814 (2)	0.09706 (11)	0.0435 (4)	
N2	0.65025 (15)	0.3643 (2)	0.14053 (13)	0.0528 (5)	
N3	0.3344 (2)	−0.1771 (2)	0.15678 (14)	0.0612 (5)	
C1	0.30487 (17)	0.1334 (2)	0.14682 (13)	0.0401 (4)	
C2	0.25371 (19)	−0.0265 (3)	0.14295 (14)	0.0459 (5)	
C3	0.1301 (2)	−0.0513 (4)	0.12710 (16)	0.0636 (7)	
H3	0.0984	−0.1604	0.1258	0.076*	
C4	0.0545 (2)	0.0879 (4)	0.11328 (18)	0.0731 (8)	
H4	−0.0291	0.0735	0.1026	0.088*	
C5	0.1024 (2)	0.2479 (4)	0.11526 (19)	0.0728 (8)	
H5	0.0507	0.3418	0.1050	0.087*	
C6	0.2264 (2)	0.2709 (3)	0.13230 (16)	0.0558 (6)	
H6	0.2577	0.3803	0.1341	0.067*	
C7	0.43942 (17)	0.1743 (2)	0.17127 (14)	0.0433 (5)	
C8	0.60936 (17)	0.2138 (3)	0.10694 (13)	0.0441 (5)	
C9	0.7795 (2)	0.4114 (3)	0.16004 (17)	0.0607 (6)	
H9A	0.8292	0.3093	0.1710	0.073*	
H9B	0.8039	0.4801	0.2156	0.073*	
C10	0.8020 (3)	0.5099 (4)	0.0808 (2)	0.0793 (8)	
H10A	0.7897	0.4366	0.0282	0.119*	
H10B	0.8844	0.5518	0.0987	0.119*	
H10C	0.7461	0.6038	0.0651	0.119*	
C11	0.5729 (2)	0.5026 (3)	0.1556 (2)	0.0737 (8)	
H11A	0.4905	0.4853	0.1175	0.111*	
H11B	0.6028	0.6090	0.1400	0.111*	
H11C	0.5747	0.5042	0.2193	0.111*	
H1A	0.4498 (18)	0.123 (3)	0.0494 (11)	0.051 (6)*	

### Atomic displacement parameters (Å^2^)

**Table:**

	*U*^11^	*U*^22^	*U*^33^	*U*^12^	*U*^13^	*U*^23^
S1	0.0395 (3)	0.0708 (4)	0.0647 (4)	0.0004 (2)	0.0194 (3)	−0.0241 (3)
O1	0.0506 (9)	0.0857 (12)	0.0461 (9)	−0.0004 (8)	0.0142 (7)	−0.0123 (8)
O2	0.0695 (12)	0.0584 (11)	0.1181 (16)	0.0213 (9)	0.0318 (11)	0.0114 (10)
O3	0.134 (2)	0.0423 (10)	0.1218 (18)	−0.0117 (11)	0.0429 (15)	−0.0064 (10)
N1	0.0362 (8)	0.0513 (10)	0.0453 (9)	−0.0046 (7)	0.0156 (7)	−0.0126 (8)
N2	0.0415 (9)	0.0572 (11)	0.0618 (11)	−0.0084 (8)	0.0185 (8)	−0.0138 (9)
N3	0.0866 (15)	0.0377 (10)	0.0639 (12)	0.0017 (10)	0.0295 (11)	0.0017 (8)
C1	0.0402 (10)	0.0417 (10)	0.0428 (10)	0.0035 (8)	0.0190 (8)	−0.0007 (8)
C2	0.0504 (11)	0.0470 (11)	0.0449 (11)	0.0001 (9)	0.0209 (9)	0.0002 (8)
C3	0.0608 (14)	0.0764 (17)	0.0602 (14)	−0.0244 (13)	0.0279 (12)	−0.0070 (12)
C4	0.0389 (12)	0.118 (2)	0.0658 (16)	−0.0025 (14)	0.0213 (11)	−0.0029 (15)
C5	0.0510 (14)	0.090 (2)	0.0805 (18)	0.0280 (14)	0.0242 (12)	0.0086 (14)
C6	0.0536 (12)	0.0485 (12)	0.0698 (14)	0.0113 (10)	0.0252 (11)	0.0041 (10)
C7	0.0405 (10)	0.0429 (11)	0.0497 (11)	0.0049 (8)	0.0181 (9)	−0.0035 (8)
C8	0.0367 (10)	0.0560 (12)	0.0400 (10)	−0.0039 (9)	0.0122 (8)	−0.0071 (8)
C9	0.0448 (12)	0.0725 (15)	0.0638 (14)	−0.0165 (11)	0.0145 (10)	−0.0187 (12)
C10	0.0656 (16)	0.0856 (19)	0.090 (2)	−0.0134 (14)	0.0287 (14)	−0.0004 (16)
C11	0.0675 (16)	0.0533 (14)	0.107 (2)	−0.0033 (12)	0.0356 (15)	−0.0200 (14)

### Geometric parameters (Å, °)

**Table:**

S1—C8	1.674 (2)	C3—H3	0.9300
O1—C7	1.206 (2)	C4—C5	1.370 (4)
O2—N3	1.212 (3)	C4—H4	0.9300
O3—N3	1.212 (3)	C5—C6	1.378 (3)
N1—C7	1.372 (2)	C5—H5	0.9300
N1—C8	1.412 (2)	C6—H6	0.9300
N1—H1A	0.849 (10)	C9—C10	1.514 (4)
N2—C8	1.319 (3)	C9—H9A	0.9700
N2—C11	1.462 (3)	C9—H9B	0.9700
N2—C9	1.469 (3)	C10—H10A	0.9600
N3—C2	1.479 (3)	C10—H10B	0.9600
C1—C2	1.381 (3)	C10—H10C	0.9600
C1—C6	1.382 (3)	C11—H11A	0.9600
C1—C7	1.509 (3)	C11—H11B	0.9600
C2—C3	1.378 (3)	C11—H11C	0.9600
C3—C4	1.372 (4)		
			
C7—N1—C8	122.31 (16)	C1—C6—H6	119.6
C7—N1—H1A	118.6 (15)	O1—C7—N1	124.04 (18)
C8—N1—H1A	113.3 (15)	O1—C7—C1	121.22 (17)
C8—N2—C11	124.45 (18)	N1—C7—C1	114.38 (17)
C8—N2—C9	121.75 (18)	N2—C8—N1	115.81 (17)
C11—N2—C9	113.66 (19)	N2—C8—S1	125.42 (15)
O3—N3—O2	124.6 (2)	N1—C8—S1	118.75 (15)
O3—N3—C2	117.3 (2)	N2—C9—C10	111.5 (2)
O2—N3—C2	118.12 (18)	N2—C9—H9A	109.3
C2—C1—C6	117.29 (18)	C10—C9—H9A	109.3
C2—C1—C7	126.44 (17)	N2—C9—H9B	109.3
C6—C1—C7	116.16 (18)	C10—C9—H9B	109.3
C3—C2—C1	122.5 (2)	H9A—C9—H9B	108.0
C3—C2—N3	118.5 (2)	C9—C10—H10A	109.5
C1—C2—N3	118.97 (18)	C9—C10—H10B	109.5
C4—C3—C2	118.9 (2)	H10A—C10—H10B	109.5
C4—C3—H3	120.6	C9—C10—H10C	109.5
C2—C3—H3	120.6	H10A—C10—H10C	109.5
C5—C4—C3	120.0 (2)	H10B—C10—H10C	109.5
C5—C4—H4	120.0	N2—C11—H11A	109.5
C3—C4—H4	120.0	N2—C11—H11B	109.5
C4—C5—C6	120.5 (2)	H11A—C11—H11B	109.5
C4—C5—H5	119.8	N2—C11—H11C	109.5
C6—C5—H5	119.8	H11A—C11—H11C	109.5
C5—C6—C1	120.9 (2)	H11B—C11—H11C	109.5
C5—C6—H6	119.6		
			
C6—C1—C2—C3	−1.2 (3)	C8—N1—C7—O1	8.5 (3)
C7—C1—C2—C3	174.71 (19)	C8—N1—C7—C1	−178.37 (17)
C6—C1—C2—N3	179.13 (19)	C2—C1—C7—O1	−95.5 (3)
C7—C1—C2—N3	−5.0 (3)	C6—C1—C7—O1	80.5 (3)
O3—N3—C2—C3	5.7 (3)	C2—C1—C7—N1	91.2 (2)
O2—N3—C2—C3	−172.8 (2)	C6—C1—C7—N1	−92.9 (2)
O3—N3—C2—C1	−174.6 (2)	C11—N2—C8—N1	−8.5 (3)
O2—N3—C2—C1	6.9 (3)	C9—N2—C8—N1	176.06 (19)
C1—C2—C3—C4	1.0 (3)	C11—N2—C8—S1	170.1 (2)
N3—C2—C3—C4	−179.3 (2)	C9—N2—C8—S1	−5.4 (3)
C2—C3—C4—C5	0.1 (4)	C7—N1—C8—N2	−63.8 (3)
C3—C4—C5—C6	−0.9 (4)	C7—N1—C8—S1	117.53 (18)
C4—C5—C6—C1	0.6 (4)	C8—N2—C9—C10	96.1 (3)
C2—C1—C6—C5	0.4 (3)	C11—N2—C9—C10	−79.8 (3)
C7—C1—C6—C5	−175.9 (2)		

### Hydrogen-bond geometry (Å, °)

**Table:**

*D*—H···*A*	*D*—H	H···*A*	*D*···*A*	*D*—H···*A*
N1—H1A···S1^i^	0.85 (2)	2.55 (2)	3.3828 (18)	167.(2)
C6—H6···O3^ii^	0.93	2.41	3.317 (3)	164

Symmetry codes: (i) −*x*+1, −*y*, −*z*; (ii) *x*, *y*+1, *z*.
